# The Women's Congress

**Published:** 1899-07-08

**Authors:** 


### The Women's Congress.

We must confess to a feeling of disappointment that English nursing has not made a better show than it has done in that fair Parliament which has lately been engaged in discussing questions of every shade connected with the interests and the prospects of women workers. Taken altogether the meeting has been a remarkable demonstration of the progress which has been made by women, if that movement is to be called progress which has chiefly consisted in the undertaking of new kinds of work. Nursing has seemed to us to stand not only on a separate but on a higher platform than much else that has been discussed, for nursing has always been woman's work, and, so different from the doctoring, and the journalism, and the hundred and one new ways now open to women anxious to earn a living, woman's progress in the field of nursing has not been a mere spreading out into new fields, but a real progress to something better and more perfect in her own field than ever existed before. And England has taken a large—perhaps almost a predominant part—in the progress that has been made. Hence our regret; for, although nursing as it exists in many nations, including our own, was represented at the Congress, English nurses and English training schools and the matrons of the great English hospitals failed to exercise that preponderating influence in its councils which might have been expected from the very great part which they have taken in elevating nursing to its present position.

Whether the position which nursing as a "profession" has attained, and still more, whether the status aimed at by many nurses, and indeed arrogated as their right by some, is an altogether healthy position or a status quite proper and appropriate to the work which a nurse has to do, may be another question. But this is clear, that what nurses need at present far more than any organisation to advance their position and their imagined interests, is some recognised head, some responsible guide, some person or body who can speak with an authority which none can question as to what is right or wrong, what is ethically correct, and what is impossible and not to be tolerated. And this brings us back again to the subject of the congress, for among the various results of that intercourse of mind with mind which such a congress brings about, has been the suggestion of an international board, or council, or committee—call it by what name one likes—which shall not only form a link among the nations but shall represent all that is best in the aspirations and in the traditions by which those engaged in nursing work are animated. But how is such a board to be organised? and how is it to be representative? In regard to the latter we can have no doubt that it should very largely, if not entirely, consist of the matrons of the great training hospitals. We have to look at facts, and the great fact which dominates the rest, and which makes it especially difficult, may be impossible, for nurses to govern themselves by any elected body, is that it is next to impossible to define the electorate. The nursing "profession" has grown out of, and is surrounded by, a vast amount of nursing which is in no true sense professional; trained nursing in its very origin came from amateurs, and it is still surrounded by a fringe of amateurs into which it fades by imperceptible degrees. But the best and the highest type is always that which is most directly in touch with the great training hospitals; it is from them that the high traditions as well as the manual training spring; it is to the matrons and thesisters of these great training schools that the alumnæ, to use the modern phraseology, turn for counsel and advice; and if a council or a board could be formed from among these matrons—people of undoubted position and stability—to whom nurses from other schools could also turn for authoritative pronouncements on serious questions as they may arise, their verdicts would be respected.

As to the question who should initiate such a plan, we are not quite sure that it should spring from among the matrons themselves. That is for them to decide. If the half-dozen matrons of the great schools would set such a thing going the thing would be done. But ladies are a trifle *difficile*. With the resources and organisation of The Hospital it might also be possible to bring the matter to an issue; but we feel very strongly that the management of such an affair as this should lie among those actually engaged in training work, and that it would be far better that no one editorially connected with any paper, whether medical or nursing, should have anything to do with it. Journalism must of necessity be in touch with what is most advanced, most changeable, most evanescent; but if the proposed international council is to be of the slightest service to the nursing profession it must be connected with all that is most stable and most staid. What nurses want is not a new parliament of tittle-tattle, not an association for the reading of papers, not an institution for the brewing of storms in tea-pots, but a voice to which they will willingly give obedience, and to which the public will give respect.

